# Micro-RNA signatures in monozygotic twins discordant for congenital heart defects

**DOI:** 10.1371/journal.pone.0226164

**Published:** 2019-12-05

**Authors:** Masood Abu-Halima, Josephin Weidinger, Martin Poryo, Dominic Henn, Andreas Keller, Eckart Meese, Hashim Abdul-Khaliq

**Affiliations:** 1 Institute of Human Genetics, Saarland University, Homburg/Saar, Germany; 2 Department of Pediatric Cardiology, Saarland University Medical Center, Homburg/Saar, Germany; 3 Department of Hand, Plastic and Reconstructive Surgery, BG Trauma Center Ludwigshafen, University of Heidelberg, Ludwigshafen, Germany; 4 Chair for Clinical Bioinformatics, Saarland University, Saarbruecken, Germany; Tufts Medical Center Molecular Cardiology Research Institute, UNITED STATES

## Abstract

**Background:**

MicroRNAs (miRNAs) are small RNAs regulating gene expression post-transcriptionally. Recent studies demonstrated that miRNAs are involved in the development of congenital heart defects (CHD). In this study, we aimed at identifying the specific patterns of miRNAs in blood of monozygotic twin pairs discordant for CHD and to assess whether miRNAs might be involved in the development or reflect the consequences of CHD.

**Methods:**

miRNA microarray analysis and Real-Time Quantitative PCR (RT-qPCR) were employed to determine the miRNA abundance level from 12 monozygotic twins discordant for CHD and their non-CHD co-twins (n = 12). Enrichment analyses of altered miRNAs were performed using bioinformatics tools.

**Results:**

Compared with non-CHD co-twins, profiling analysis indicated 34 miRNAs with a significant difference in abundance level (p<0.05, fold change ≥ 1.3), of which 11 miRNAs were up-regulated and 23 miRNAs were down-regulated. Seven miRNAs were validated with RT-qPCR including miR-511-3p, miR-1306-5p, miR-421, miR-4707-3p, miR-4732-3p, miR-5189-3p, and miR-890, and the results were consistent with microarray analysis. Five miRNAs namely miR-511-3p, miR-1306-5p, miR-4732-3p, miR-5189-3p, and miR-890 were found to be significantly up-regulated in twins < 10 years old. Bioinformatics analysis showed that the 7 validated miRNAs were involved in phosphatidylinositol signaling, gap junction signaling, and adrenergic signaling in cardiomyocytes.

**Conclusions:**

Our data show deregulated miRNA abundance levels in the peripheral blood of monozygotic twins discordant for CHD, and identify new candidates for further analysis, which may contribute to understanding the development of CHD in the future. Bioinformatics analysis indicated that the target genes of these miRNAs are likely involved in signaling and communication of cardiomyocytes.

## Introduction

During the last two centuries, the percentage of twin births in the general population has increased. Assisted reproductive technologies are likely responsible for the increased number of twin births because multiple embryos are implanted [1, 2]. In general, the rate of congenital anomalies is higher in twins compared with singletons [3–5], especially the discordance for cardiovascular anomalies [3, 6]. Moreover, it has been shown that the risk of congenital anomalies among twins that share a placenta (i.e. monochorionic twins, who are almost uniformly monozygotic) is nearly twice as high compared to twins that do not share a placenta (i.e. dichorionic, mostly dizygotic twins) [3].

Congenital heart defect (CHD) is one of the most common types of birth defects, with a neonatal incidence of 0.8–1.0% [7]. CHD includes a broad range of heart development malformations that begin early in fetal development such as atrial septal defects (ASD), ventricular septal defects (VSD), Tetralogy of Fallot [8], and transposition of the great arteries [9]. The clinical spectrum of congenital heart defects is broad from mild to severe and complex, such as ASDs and VSDs being usually clinically asymptomatic until school age or early adulthood, while others such as hypoplastic left heart syndrome, tetralogy of Fallot, and transposition of great arteries lead to cyanosis and inadequate pulmonary and systemic perfusion if adequate surgical treatment is not performed [7]. Accordingly, the clinical course may range from spontaneous recovery up to a life-long medical treatment with multiple interventional and surgical procedures, often already starting in the early postnatal age.

Over the last years, it has become apparent that a wide variety of protein-coding genes and other molecules such as non-coding RNAs are involved in maintaining a restricted and specific pattern of gene expression in the heart [10]. MiRNAs are a class of non-coding RNAs of approximately 22 nucleotides in length that regulate gene expression post-transcriptionally via a sequence-specific interaction with the 3' UTR of target mRNAs, resulting in inhibition of translation and/or mRNA degradation [11]. Since a non-CHD co-twin in a CHD-discordant monozygotic twin pair provides a well-matched control, we hypothesized that alterations in miRNA abundance level might contribute to the discordance in monozygotic twin pairs with CHDs. To test this hypothesis, we investigated twelve monozygotic twin pairs which were discordant for CHD using high-throughput microarray screening and RT-qPCR validation analysis.

## Methods

### Patients and sample collection

The study was conducted following the Declaration of Helsinki and was approved by the locally appointed ethics committee (Ethik-Kommission Landesärztekammer des Saarlandes, No. 178/14). A total of 12 twin pairs (14 females, 10 males) with a mean age of 11.00 ± 5.53 years were enrolled in the study (**Table 1**). Only twin pairs in which one co-twin was diagnosed with CHD and the other co-twin was healthy (non-CHD) were included in the study. None of the patients at the time of blood collection was diagnosed with heart failure and all included twins were asymptomatic without heart failure. The majority had already undergone corrective, palliative cardiac surgery or therapeutic catheter intervention, while others were still in follow up because therapeutic approaches were still not necessary. In this study, twins with ASD and VSD were only considered, when they hemodynamically effective and only patients with hemodynamically effective PDA, who need interventional closure were also considered. All twin patients were still under medical treatment and follow up at the Department of Pediatric Cardiology at the University Hospital of Saarland, Homburg/Saar during data collection. Clinical data were collected from the patient charts and the twins' parents. Venous blood from the cubital vein was drawn and 2.5 mL were collected in PAXgene^TM^ blood tubes (BD Biosciences, San Jose, CA, USA) and stored at room temperature for 2 hours till complete lysis of blood cells was achieved before they were stored at -20°C until RNA including miRNA isolation. Another 2.5 mL blood sample was collected from each included subject for the DNA typing. All samples were collected and processed at the Department of Pediatric Cardiology and the Institute for Human Genetics at the University Hospital of Saarland, Germany. Samples were analyzed according to standard operating procedures after all participants or their legal guardians had given their written informed consent.

**Table 1:** Characteristics of the monozygotic twins included in the study.

Code	Age	Gender	CHD
HOAZ_1003	7	Male	AVSD
HOAZ_1004	7	Male	non-CHD
HOAZ_1005	5	Male	MI, LVOTO, PDA, AS
HOAZ_1006	5	Male	non-CHD
HOAZ_1010	17	Female	PDA
HOAZ_1011	17	Female	non-CHD
HOAZ_1012	11	Male	DILV, VSD
HOAZ_1013	11	Male	non-CHD
HOAZ_1015	5	Female	CoA
HOAZ_1016	5	Female	non-CHD
HOAZ_1019	5	Female	LPA-Stenosis
HOAZ_1020	5	Female	non-CHD
HOAZ_1023	10	Male	ASD II
HOAZ_1024	10	Male	non-CHD
HOAZ_1027	19	Female	non-CHD
HOAZ_1028	19	Female	Coronary fistula
HOAZ_1033	12	Female	TOF, VSD, ASD
HOAZ_1034	12	Female	non-CHD
HOAZ_1035	9	Female	VSD
HOAZ_1036	9	Female	non-CHD
HOAZ_1039	22	Female	non-CHD
HOAZ_1040	22	Female	mid-aortic syndrome
HOAZ_1041	10	Male	bicuspid aortic valve
HOAZ_1042	10	Male	non-CHD

CHD, congenital heart disease; AVSD, Atrioventricular septal defect; MI, Mitral insufficiency; LVOTO, Left ventricular Outflow Tract Obstruction; PDA, Patent Ductus arteriosus; AS, Aortic stenosis; TGA, Transposition of the great arteries; ASD, Atrial septal defect; VSD, Ventricular septal defect; TOF, Tetralogy of Fallot; DILV, Double inlet left ventricle; CoA, Coarctation of the aorta; LPA-Stenosis, Left pulmonary artery stenosis; PS, Pulmonary stenosis

### Zygosity determination

Zygosity of the twin pairs was tested using the AmpFιSTR^®^ Identifiler^™^/NGM SElect^™^ PCR Amplification Kit (Applied Biosystems, Foster City, CA, USA) at the Institute of Forensic Medicine, Charité University of Berlin (**S1 Table**). Briefly, genomic DNA was extracted from the blood of each participant using the ReliaPrep^™^ Large Volume HT gDNA Isolation System (Promega, Mannheim, Germany). The DNA integrity, purity, and concentration were evaluated by agarose gel (0.8%) electrophoresis and by spectrophotometry at the wavelengths of 230 nm, 260 nm, and 280 nm. Zygosity of the twin pairs was confirmed by typing all included twins with the 22 Short Tandem Repeat loci namely D10S1248, vWA, D16S539, D2S1338, D8S1179, D21S11, D18S51, D22S1045, D19S433, TH01, FGA, D2S441, D3S1358, D1S1656, D12S391, SE33, D7S820, CSF1PO, D13S317, TPOX and D5S818. PCR products were separated on an ABI capillary sequencer and evaluated using GeneMapper ID Software (Applied Biosystems).

### Isolation of total RNA, including miRNAs

Total RNA, including miRNAs of all patients and their non-CHD co-twins, was extracted with the PAXgene miRNA blood kit using the QIAcube^™^ automated isolation instrument according to the manufacturer's instructions (Qiagen, Hilden, Germany). The concentration and purity of the samples were measured using the NanoDrop ND-1000 Spectrophotometer (Thermo Fisher Scientific, Massachusetts, USA). The RNA integrity was assessed with an Agilent 2100 Bioanalyzer using RNA 6000 Nano kit (Agilent Technologies, California, USA). DNase I (Thermo Fisher Scientific, Massachusetts, USA) treatment was carried out according to the manufacturer’s instructions to remove any DNA contamination. Conventional PCR with exon spanning primers for Glyceraldehyde 3-phosphate dehydrogenase (GAPDH) was performed to exclude residual DNA in the samples as previously described [12].

### miRNA profiling

miRNA profiling analysis of 12 twin pairs (24 individuals) was carried out using SurePrint G3 Human miRNA, 8X60K microarray, covering 2549 human miRNAs (Sanger miRBase release 21) (Agilent Technologies). These microarrays contain ~20 replicates for each probe complement to each of the 2549 mature miRNAs of miRBase v21. These probes act in concert to measure the miRNA of interest, and the data are combined later during software analysis. All probes are randomly distributed on the array, and cross-hybridization is prevented by the addition of a G residue and a hairpin at the 50-end of the probe. All procedures were carried out as previously described [13, 14]. In brief, a total of 100 ng total RNA from each sample was dephosphorylated by incubation with calf intestinal phosphatase at 37°C for 30 minutes and denatured with the use of 100% dimethyl sulfoxide at 100°C for 7 minutes. Samples were labeled with pCp-Cy3 with the use of T4 ligase at 16°C incubation for 2 hours. Each labeled RNA sample was hybridized onto an individual sub-array of the 8×60K format Agilent miRNA microarray slide. Then the microarrays were loaded and incubated at 55°C for 20 hours with rotation. After two washing steps, the arrays were dried and scanned using the Agilent Microarray Scanner at 3 microns in double path mode. Data was acquired using Agilent AGW Feature Extraction software version 10.10.11 (Agilent Technologies).

### Analysis of miRNAs by RT‑qPCR

RT-qPCR validation analysis was performed according to the manufacturer’s instructions using *the mi*Script PCR System (Qiagen). Complementary DNA (cDNA) was generated by reverse transcription of 200 ng of total RNA using the *mi*Script RT II Kit (Qiagen). Briefly, 200 ng of total RNA containing miRNAs was mixed with 4 μL miScript HiSpec Buffer, 2 μL nucleic mix, 2 μL *mi*Script Reverse Transcriptase mix and PCR grade water to a final volume of 20 μL. Following the reverse transcription reaction, the cDNA was diluted 1:10. Two-μL of diluted cDNA was mixed with 10 μL *mi*Script SYBR Green PCR Master Mix, 2 μL *mi*Script Universal Primer, 2 μL *mi*Script Primer Assay for eleven miRNAs (miR-511-3p, miR-1306-5p, miR-421, miR-134-3p, miR-4707-3p, miR-4732-3p, miR-1281, miR-6511a-3p, miR-5189-3p, miR-943, miR-890) and RNU6B snRNA as an endogenous control (Qiagen) in a total volume of 20 μL. All RT-qPCR experiments were performed using the QIAgility^™^ automated PCR setup robot (Qiagen) before performing RT-qPCR analysis in the StepOnePlus^™^ Real-Time PCR system (Applied Biosystems) as previously described [15]. The melting curve analysis was used to control the specificity of RT-qPCR products. The specificity of amplicons was further confirmed by agarose gel electrophoresis.

### Statistical analysis

R statistical environment v.2.14.2 was used to analyze the differences in miRNA abundance level in patients and their non-CHD co-twins. The intensity values of miRNAs were extracted with the use of Agilent Feature Extraction image analysis software. To compute the total abundance level per miRNA and sample, we summed up the gTotalProbeSignals. A quantile normalization method was applied to normalize abundance levels across the arrays with the use of the preprocess Core package of the R programming language. The significance level of each miRNA was then analyzed by applying student t-test and area under the receiver operating characteristic curve (AUC) values for each miRNA were computed. For validation of the microarray results, we used the relative quantitative method of 2^−ΔΔCt^ [16] to measure the differences of miRNA abundance between the two tested groups i.e. patients with CHD and their non-CHD co-twins. Results were statistically analyzed with GraphPad Prism (version 7.04, GraphPad Software Inc., San Diego, CA) and presented as mean ± SD. Statistically significant differences in the level of each miRNA between the two paired twins were assessed by paired t-test. *P-*values <0.05 were considered statistically significant.

### Target prediction and functional analysis

Enriched KEGG pathway analyses were performed using DIANA-miRPath v.3.0 software which utilizes predicted miRNA targets (in the coding sequence (CDS) or 3ˋ-UTR regions) provided by DIANA-microT-CDS algorithm [17]. Targets of miRNAs with a threshold of more than 0.8 were selected. Only KEGG pathways with *P*-values <0.05 and a false discovery rate (FDR) <0.05 were retained. The effect of miRNAs on target genes and networks has been evaluated using miRTargetLink software [18].

## Results

### Differentially abundant miRNAs

We found 208 (8.16%) miRNAs with a significant difference in abundance level when comparing twins with CHD to their non-CHD co-twins **(S2 Table)** (p<0.05). When considering the differentially abundant miRNAs a fold change of ≥ 1.3 in the twins with CHD compared to their non-CHD co-twins, 34 miRNAs (1.33%) were found to be differentially abundant, of which 11 miRNAs were up-regulated and 23 miRNAs were down (**Table 2**) (P <0.05). As shown in **Table 2**, the mean fold change of the up-regulated miRNAs was 1.52 ± 0.30, whereas the mean fold change of the down-regulated was 0.71 ± 0.05. Eleven miRNAs were chosen for further validation by RT-qPCR based on their differential abundance level as determined by microarray between twins with CHD and their non-CHD co-twins and based on their known associations with cardiovascular diseases. In detail, we selected four miRNAs with highest (miR-5189-3p, miR-4707-3p, miR-1306-5p, and miR-4732-3p) and three moderate (miR-511-3p, miR-890, and miR-421) fold change among the up-regulated miRNAs and two miRNAs with highest (miR-943 and miR-6511a-3p) and moderate (miR-1281 and miR-134-3p) fold change among the down-regulated miRNAs. In addition, we selected five miRNAs (miR-1306-5p, miR-511-3p, miR-421, miR-1281, and miR-134-3p) with higher and moderate or low abundance levels based on the array analysis and with known association with cardiac pathologies, vascular remodeling, aortic aneurysm and its related process [14, 15, 19–25].

**Table 2:** Significantly abundant miRNAs in the blood of monozygotic twin pairs with CHD (n = 12) compared to their non-CHD co-twins (n = 12) as determined by microarray analysis (P <0.05).

miRNAs	Median non-CHD	Median CHD	STDV non-CHD	STDV CHD	Fold Change	Regulation	P-value	AUC
miR-5189-3p	4.33	10.39	3.48	4.01	2.38	Up	0.0176	0.77
miR-4707-3p	2.64	4.42	1.37	1.81	1.67	Up	0.0208	0.66
miR-1306-5p	4.73	7.63	2.30	2.81	1.61	Up	0.0206	0.67
miR-4732-3p	34.81	53.13	25.02	36.38	1.52	Up	0.0102	0.64
miR-3605-3p	3.62	5.22	1.19	1.42	1.45	Up	0.0447	0.66
miR-550b-2-5p	5.62	7.69	2.22	2.15	1.37	Up	0.0228	0.65
miR-511-3p	0.83	1.13	0.23	0.29	1.35	Up	0.0329	0.73
miR-890	0.87	1.17	0.28	0.18	1.35	Up	0.0309	0.81
miR-671-5p	2.47	3.31	0.95	1.16	1.33	Up	0.0385	0.69
miR-128-1-5p	2.10	2.82	0.81	0.73	1.35	Up	0.0483	0.72
miR-421	2.54	3.35	0.76	0.67	1.32	Up	0.0193	0.72
miR-7109-3p	4.28	2.37	2.27	1.63	0.56	Down	0.0278	0.38
miR-943	2.07	1.28	0.64	0.42	0.62	Down	0.0038	0.24
miR-6511a-3p	4.38	2.76	4.38	1.52	0.63	Down	0.0323	0.30
miR-6735-3p	2.73	1.81	1.66	0.72	0.67	Down	0.0329	0.24
miR-6787-3p	3.15	2.12	2.37	1.41	0.67	Down	0.0133	0.35
miR-6877-3p	4.13	2.82	1.30	0.51	0.68	Down	0.0090	0.23
miR-4296	1.50	1.04	0.44	0.51	0.69	Down	0.0459	0.36
miR-1281	7.78	5.35	16.10	9.02	0.69	Down	0.0299	0.33
miR-3677-3p	1.18	0.84	0.40	0.19	0.71	Down	0.0083	0.24
miR-4763-5p	3.68	2.66	1.71	1.05	0.72	Down	0.0360	0.36
miR-7106-3p	2.40	1.76	0.96	0.50	0.73	Down	0.0086	0.23
miR-6789-3p	2.54	1.85	0.91	0.63	0.73	Down	0.0366	0.35
miR-1825	7.09	5.19	13.02	6.84	0.73	Down	0.0481	0.35
miR-134-3p	2.43	1.78	0.56	0.36	0.74	Down	0.0064	0.26
miR-1343-3p	2.42	1.80	0.89	0.71	0.74	Down	0.0307	0.33
miR-6727-3p	2.08	1.55	0.57	0.66	0.75	Down	0.0033	0.29
miR-6736-3p	3.14	2.34	0.84	0.56	0.75	Down	0.0083	0.30
miR-8075	1.59	1.20	0.57	0.29	0.75	Down	0.0241	0.28
miR-3714	2.66	2.02	1.02	0.63	0.76	Down	0.0043	0.21
miR-6879-3p	2.66	2.02	1.04	0.79	0.76	Down	0.0250	0.38
miR-675-3p	2.42	1.84	0.67	0.69	0.76	Down	0.0292	0.27
miR-6743-3p	5.02	3.81	13.28	8.08	0.76	Down	0.0312	0.37
miR-4800-3p	2.24	1.72	0.60	0.43	0.76	Down	0.0158	0.32

Each value represents the median abundance value of each tested group ± standard deviation (STDV). CHD, Congenital heart defects; AUC, area under the receiver operating characteristic curve.

### Validation of candidate miRNAs by RT-qPCR

**Fig 1:**
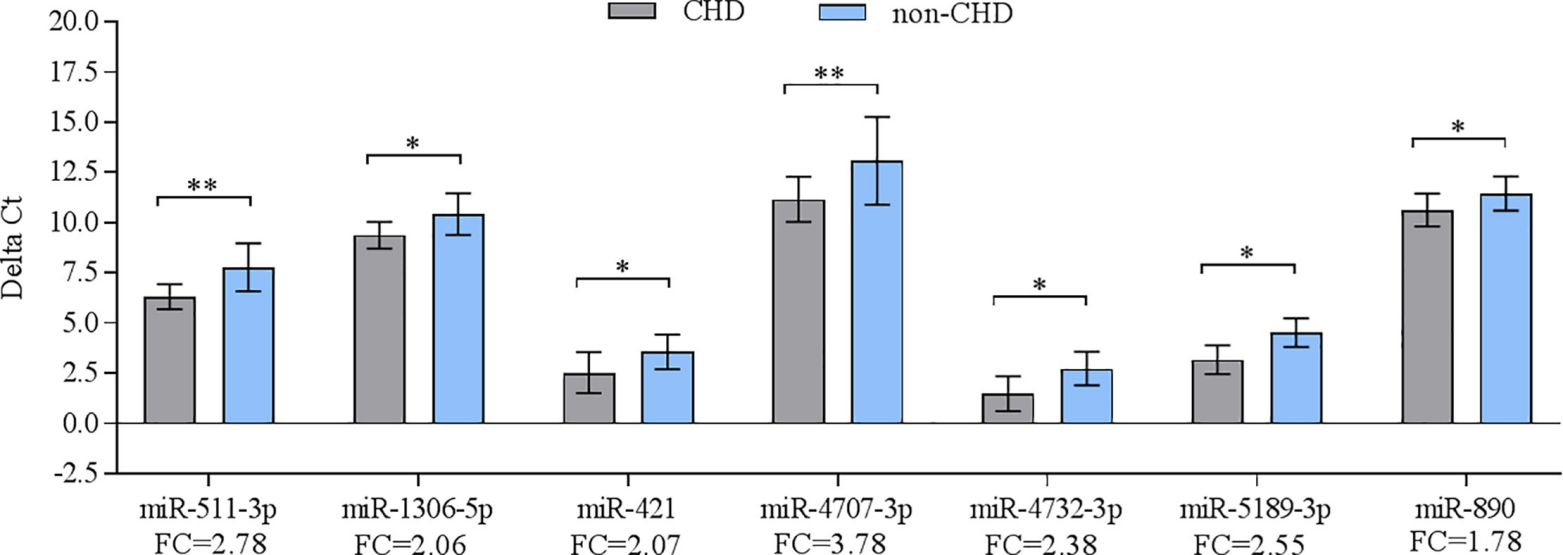
Validation of differentially abundant miRNAs in the blood of monozygotic twin pairs with CHD (n = 12) compared to their non-CHD co-twins (n = 12) as determined by RT-qPCR analysis (P < 0.05). Mean ΔCt of each group were considered (lower ΔCt, higher abundance level). RNAU6B as an endogenous control for normalization, unpaired t-tests and ±standard deviation (STDV) were used to evaluate differences in abundance level. * P ≤ 0.05; ** P ≤ 0.01; *** P ≤ 0.001.

**Fig 2:**
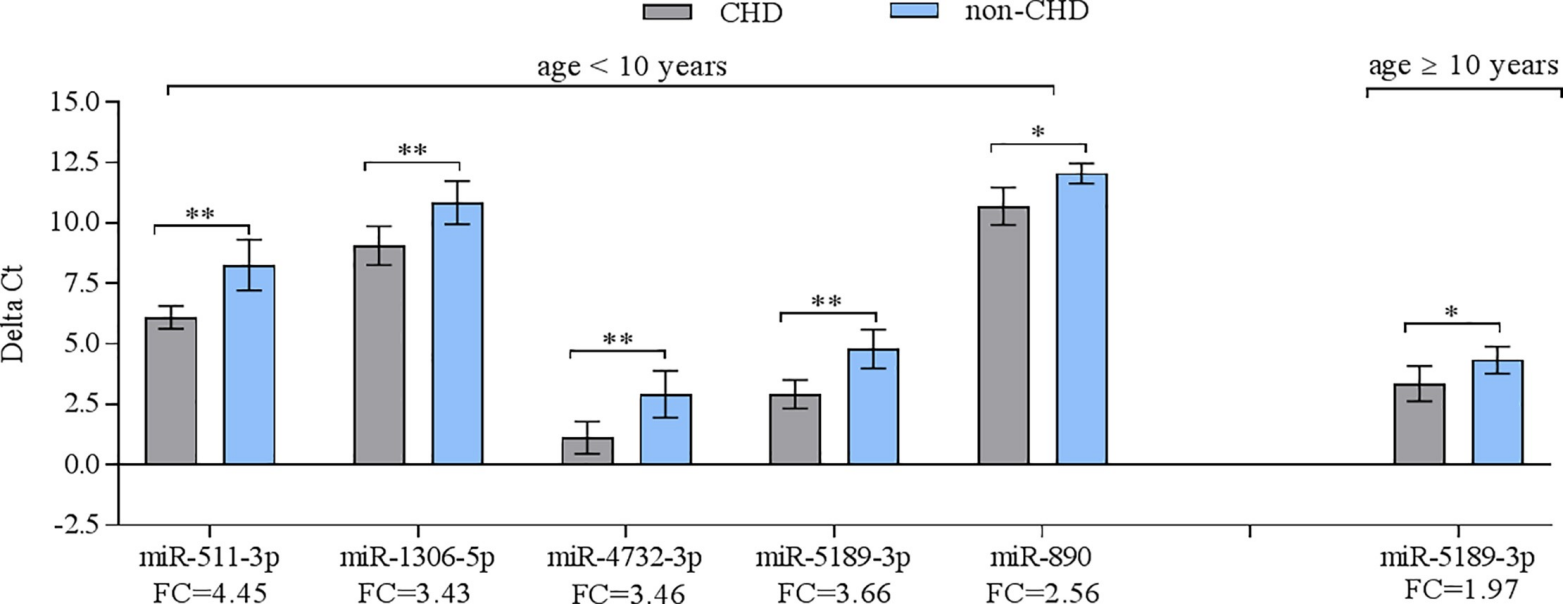
Validation of differentially abundant miRNAs in the blood of monozygotic twin pairs with CHD compared to their healthy co-twins according to age [10 ≥ years (n = 7 twin pairs) and 10< years (n = 5 twin pairs) as determined by RT-qPCR analysis (P < 0.05). Mean ΔCt of each group were considered (lower ΔCt, higher abundance level). RNAU6B as an endogenous control for normalization, unpaired t-tests and ±standard deviation (STDV) were used to evaluate differences in abundance level. * P ≤ 0.05; ** P ≤ 0.01; *** P ≤ 0.001.

Using RT-qPCR, the relative abundance level of 11 miRNAs was confirmed in the same samples, which have been used for the microarray experiments. As shown in **Fig 1,** RT-qPCR showed the same direction of abundance changes as the microarray analysis for 7 out of 11 used miRNAs when comparing the samples from patients with CHD to their non-CHD co-twins (miR-511-3p, miR-1306-5p, miR-421, miR-4707-3p, miR-4732-3p, miR-5189-3p, and miR-890). Significant changes in abundance were confirmed for these seven up-regulated miRNAs **(**p < 0.05). No significant differences in abundance were found for two miRNAs, namely miR 6511a-3p and miR-943 (p = 0.1309 and 0.1639, respectively). In addition, miR-134-3p and miR-1281 displayed significant changes in abundance in the opposite direction in RT-qPCR compared to microarray analysis (p = 0.0108 and p = 0.0310, respectively). Next, we grouped the samples into two groups according to the median age distribution of our patients; twins younger than 10 years (< 10 years) and twins older than 10 years (≥ 10 years). As shown in **Fig 2**, we found a significant up-regulation for 5 miRNAs including miR-511-3p, miR-1306-5p, miR-4732-3p, miR-5189-3p, and miR-890 (p < 0.05) in < 10 years group. In ≥ 10 years group, there was no significance in miRNA abundance level when comparing the patients against their healthy counterparts (**Fig 2**). MiR-5189-3p showed a 1.97 fold change when comparing patients ≥10 years with their healthy co-twins, however, statistical significance was marginally missed (p = 0.0863).

### Comparative pathway analysis

**Fig 3:**
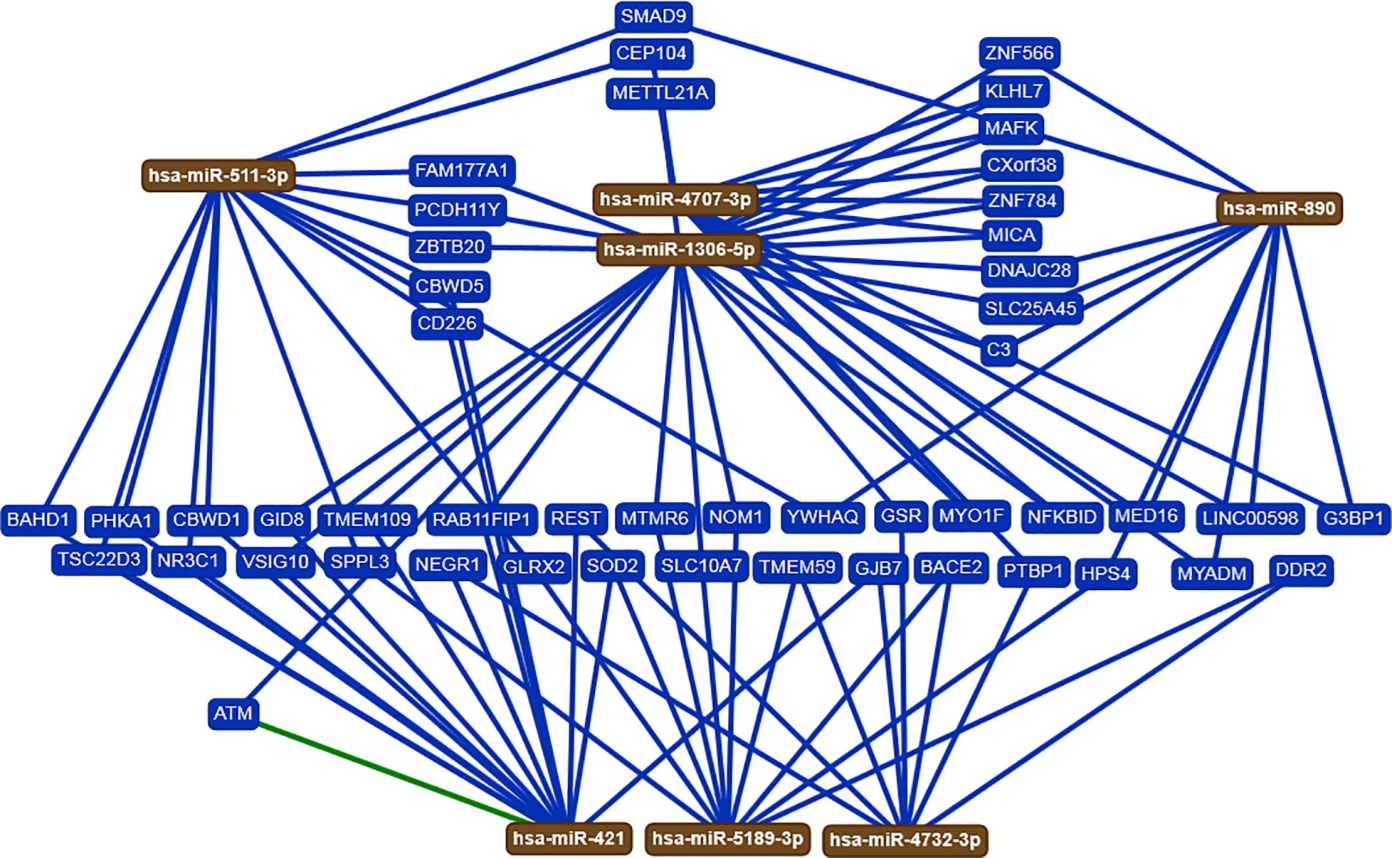
Target network of validated seven deregulated miRNAs by RT-qPCR analysis in the blood of monozygotic twin pairs with CHD (n = 12) compared to their non-CHD co-twins (n = 12) as determined by miRTargetLink algorithm. MiRNAs are shown as brown nodes. Strong and weak interactions were observed between miRNAs and genes are highlighted in ''Green'' and ''Blue'' in the resulting networks, respectively.

We used the DIANA-mirPath algorithm to gain insights into the biological pathways of the miRNAs that showed significant abundance changes between patients with CHD and their non-CHD co-twins. Based on the seven validated miRNAs by RT-qPCR (miR-511-3p, miR-1306-5p, miR-421, miR-4707-3p, miR-4732-3p, miR-5189-3p, and miR-890), we identified ten KEGG pathways that were significantly enriched (p<0.05) for targets of the validated miRNAs. As shown in **Table 3**, the target genes of the altered miRNAs are involved in signaling transduction pathways, adrenergic signaling in cardiomyocytes, cellular community, and many other pathways. Using miRTargetLink, the resulting networks are drawn schematically in **Fig 3.** The strong and weak interactions which were observed between miRNAs and genes are highlighted in the green' and blue in the networks, respectively **(Fig 3).**

**Table 3:** The KEGG pathways significantly enriched for target genes of validated seven deregulated miRNAs by RT-qPCR analysis in the blood of monozygotic twin pairs with CHD (n = 12) compared to their non-CHD co-twins (n = 12) as determined by DIANA-mirPath algorithm (P <0.05).

KEGG pathway	P-value	Gene Name	miRNA name
Gap junction (hsa04540)	4.05E^-06^	NRAS, TUBA1B, GRM5, TUBB6, EGFR, GNAI3, TJP1, PLCB1, TUBA1C, TUBB2A, PRKX, TUBB3, GUCY1A2, GJA1, MAP3K2, PRKG1, ADCY4, ADRB1,EGF, PDGFRB	miR-1306-5p, miR-890, miR-5189-3p, miR-421, miR-511-3p
Biotin metabolism (hsa00780)	4.47E^-05^	HLCS	miR-890
Morphine addiction (hsa05032)	1.30E^-02^	PDE10A GNAI3, PDE1B, GABRB3, GNB2, GNG7, PRKX, KCNJ6, KCNJ9, GABRB2, ADCY4	miR-890, miR-421, miR-1306-5p, miR-4732-3p, miR-511-3p
Glioma (hsa05214)	1.30E^-02^	CAMK2D, NRAS, CALM1, PIK3CB, EGFR, CDK6, CALM2, IGF1, MTOR, PTEN, EGF, PDGFRB	miR-890, miR-511-3p, miR-421, miR-4732-3p, miR-1306-5p
Adrenergic signaling in cardiomyocytes (hsa04261)	1.33E^-02^	CAMK2D, PPP2R5E, CALM1, PIK3CB, ATF6B, CACNB4, PPP1CC, PPP2R5D, GNAI3, PPP2R2D, PPP2R5C, CALM2, ATP2B1, CREB1, PLCB1, SLC8A1, PRKX, CACNB2, ADCY4, ADRB1, KCNE1	miR-1306-5p, miR-421, miR-890, miR-511-3p, miR-4732-5p
Dopaminergic synapse (hsa04728)	1.54E^-02^	CAMK2D, GSK3B, PPP2R5E, CALM1, ATF6B, PPP1CC, PPP2R5D, PPP3CC, GNAI3, PPP2R2D, PPP2R5C, CALM2, CREB1, MAOB, PLCB1, GNB2, SCN1A, GNG7, PRKX, KCNJ6, KCNJ9, CLOCK, GRIN2B	miR-890, miR-1306-5p, miR-511-3p, miR-421, miR-4732-3p, miR-8189-3p
Phosphatidylinositol signaling system (hsa04070)	1.64E^-02^	PLCD4, CDS2, CALM1, PIK3CB, ITPKB, IPPK, INPP4B, CALM2, PIP4K2A, PLCB1, OCRL, DGKD, PTEN, PI4K2B, DGKI, DGKH	miR-890, miR-511-3p, miR-4732-3p, miR-1306-5p, miR-5189-3p, miR-421
Oxytocin signaling pathway (hsa04921)	1.64E^-02^	CAMK2D, NRAS, PRKAA2, CALM1, PIK3CB, CACNB4, PPP1CC, ROCK2, PPP3R1, KCNJ14, CAMKK1, EGFR, PPP3CC, GNAI3, PPP1R12B, CALM2, CAMKK2, PLCB1, PRKAG1, PRKX, KCNJ6, GUCY1A2, KCNJ9, CACNB2, CAMK1D, ADCY4, MYLK	miR-511-3p, miR-421, miR-890, miR-1306-5p, miR-5189-3p, miR-4732-3p
Thyroid hormone synthesis (hsa04918)	1.98E^-02^	ATF6B, GSR, CREB1, PLCB1, PRKX, TG, ADCY4	miR-421, miR-1306-5p, miR-890
Circadian entrainment (hsa04713)	3.62E^-02^	CAMK2D, CALM1, GNAI3, CALM2, CREB1, PLCB1, GNB2, GNG7, PRKX, KCNJ6, GUCY1A2, KCNJ9, PRKG1, ADCY4, GRIN2B	miR-421, miR-890, miR-1306-3p, miR-4732-3p, miR-511-3p, miR-5189-3p

## Discussion

In this study, we identified altered miRNA abundance levels in monozygotic twins with CHDs, when compared with their non-CHD co-twins by microarray and RT-qPCR analyses. miRNA profiling analysis using microarrays indicated 34 miRNAs with differential abundance levels, of which 11 miRNAs were up-regulated and 23 miRNAs were down-regulated (P <0.05, fold change ≥1.3). Using RT-qPCR, 7 miRNAs were validated in the direction of their abundance changes. Our data indicate that the differential abundance level of these miRNAs might contribute to the development of CHD and the discordance of CHD in monozygotic twins. With rare exceptions, CHD is genetically multifactorial rather than purely inheritable disturbance of prenatal development. Between about embryonic weeks 3 to 5, a delicate concerted action of origin and clinical severity gene activities is required to properly perform the fusion of the heart tubes and the endocardial cushions, the formation of septa, cleavage of aortic and pulmonary trunks and formation of valves [26]. It is therefore well conceivable that even slight alterations of the intra-uterine microenvironment, e.g., by temporary local disturbances of maternal blood supply can cause lasting structural defects of the developing organ, in spite of the identical genomic composition of monozygotic twins. Thus, discordance for the development of structural defects in the cardiovascular system among monozygotic twins reflects the strong exogenous influences on the organogenesis of the fetal heart.

Epigenetic factors play an important role in the regulation of gene expression through multiple molecular mechanisms including binding of small molecules to specific sites in DNA like non-coding RNAs (ncRNAs) and miRNAs and serve as ‘volume controls’ that tune-up or down a gene's expression [27]. Further studies, however, should be carried out to evaluate the effects of miRNAs on epigenetic machinery and the control of miRNA expression by epigenetic mechanisms in patients of congenital heart and vessel defects. MiRNAs are of eminent importance for the regulation of gene activities in embryogenesis, in particular for the opening and closure of the often short windows of time designated for the proliferation and spatial organization of developing heart muscle, endocardial, and connective tissue cell [26, 28]. A wide variety of miRNAs are involved in the embryogenesis and development of the heart and appears likely that CHD conversely alters postnatal expression patterns of multiple miRNAs in a variable fashion. Alterations of miRNA expression patterns are unlikely a result of hemodynamic changes and decreased saturation caused by CHD. We postulate that the significant difference in the abundance of miRNAs in these twins with and without CHD may also be influenced by the congenital defects and the ongoing tissue remodeling or degeneration of the developing heart.

In this study, we identified 5 miRNAs with significant up-regulation in monozygotic twins with CHD <10 years, when compared with their non-CHD co-twins by microarray and RT-qPCR analysis. A significant up-regulation of miR-1306-5p was found in twins with CHD <10 years compared to non-CHD without CHD. This observation may be related to the accelerated growth and maturation level in young twins, which is higher during the first 10 years of age, however, it is premature to draw conclusions about the influence of age on the data. Van Boven *et al*. showed that repeatedly measured miR-1306-5p in acute heart failure patients was positively associated with a composite endpoint of all-cause mortality and re-hospitalization due to heart failure [23]. It appears likely, that an up-regulation of miR-1306-5p is mainly linked to the cardiovascular system, which also undergoes maturation and growth in this age. We found a significant up-regulation of miR-421 by RT-qPCR (Fold change: 2.07, p = 0.0264) in twins with CHD compared to their non-CHD co-twin. However, microarray analysis revealed only a marginal up-regulation (Fold change: 1.32, p = 0.0193), which may be associated with the low sample size of our study. MiR-421 is downregulated in patients with TOF and heart failure compared to TOF patients without heart failure [14]. Moreover, miR-421 is involved in mitochondrial fragmentation and cardiomyocyte apoptosis together with E2F1 and Pink1 [24] and regulates angiotensin-converting enzyme 2 (ACE2) expression. Therefore, miR-421 appears to play a critical role in early cardiac remodeling and a decreased miR-421 expression in twins with CHD in our study may be indicative of heart failure in this cohort.

To the best of our knowledge miR-511-3p, miR-890, and miR-4732-3p, which we found to be up-regulated in twins with CHD < 10 years of age, miR-5189-3p which was found to be up-regulated in twins with CHD regardless of age as well as miR-4707-3p which was up-regulated in twins with CHD in the RT-qPCR analysis, have neither been reported to be deregulated in association with cardiac disease in general nor between monozygotic twins. Our data show a differential abundance of these miRNAs in our patients and indicate that these miRNAs may be associated with the development of CHD in monozygotic twins. So far, these miRNAs have been poorly characterized and future studies analyzing their function and potential role in the development of CHD are needed.

KEGG pathway analysis revealed that the predicted target genes of the 7 validated miRNAs were involved in phosphatidylinositol signaling, gap junction signaling, and adrenergic signaling in cardiomyocytes among other pathways. Therefore, it appears likely that the pathogenic role the candidate miRNAs identified in our study play in the development of CHD, is mediated via deregulated signaling and cellular communication in cardiomyocytes.

### Study limitations

Limitations of our study are related to the limited sample size and heterogeneity of affected monocytic twins. Moreover, we had to incorporate CHD of different morphological and clinical types and severity, due to the low incidence of monozygotic twins discordant for CHD. Since it has been shown that even non-CHD monozygotic twins display a considerable variation of their miRNA fingerprints [29], we were prepared to encounter a highly complex and heterogeneous picture. It is a challenge to recruit and assay monozygotic twins and even collect data for a given phenotype. Relatedly, and critically, the reported miRNAs need to be furtherly validated in a larger and independent cohort of monozygotic twins to confirm the abundance level of the identified miRNAs. Furthermore, the precise abundance level and mechanisms underlying the identified miRNAs should be evaluated in subgroups of uniform diagnosis of congenital heart defects. Nevertheless, in our study, significant differences in the miRNA abundance level were found between the affected and non- affected monocytic twins. These differences may induce several physiological responses in the twins with CHD, which in part is reflected by changed miRNA abundance levels.

## Conclusion

We identified a set of deregulated miRNAs in monozygotic twins with CHD compared to their non-CHD co-twins. The predicted target genes of these miRNAs are involved in cardiomyocyte signaling and communication. The alteration of the abundance levels of these miRNAs provides new candidates for further analysis, which may contribute to understanding the development of CHD in the future.

## Supporting information

S1 Table Confirmation of the twin zygosity by STR typing. (DOCX)

S2 Table Differentially abundant miRNAs in the blood of monozygotic twin pairs with CHD compared to their non-CHD co-twins as determined by microarray analysis (p<0.05). (DOCX)
