# Supplementary material for: Micro-RNA signatures in monozygotic twins discordant for congenital heart defects
Source: PLoS One. 2019 Dec 5;14(12):e0226164. doi: 10.1371/journal.pone.0226164 (PMC6894838; doi:10.1371/journal.pone.0226164)
Supplement: S1 Table — (DOCX) [file pone.0226164.s001.docx]

**S1 Table:** Confirmation of the twin zygosity by STR typing

| **Code** | **D10S1248** | **vWA** | **D16S539** | **D2S1338** | **Amel.** | **D8S1179** | **D21S11** | **D18S51** | **D22S1045** | **D19S433** | **TH01** | **FGA** | **D2S441** | **D3S1358** | **D1S1656** | **D12S391** | **SE33** | **D7S820** | **CSF1PO** | **TPOX** | **D5S818** | **D13S317** |
| --- | --- | --- | --- | --- | --- | --- | --- | --- | --- | --- | --- | --- | --- | --- | --- | --- | --- | --- | --- | --- | --- | --- |
| HOAZ_1003 | 14 | 17,19 | 11,12 | 19,2 | X,Y | 14 | 30 | 14,18 | 15 | 13,15 | 7,8 | 20,23 | 10,11 | 15,16 | 11,17.3 | 18.3,21 | 21,23.2 | 10,12 | 11 | 8,12 | 11,12 | 8,14 |
| HOAZ_1004 | 14 | 17,19 | 11,12 | 19,2 | X,Y | 14 | 30 | 14,18 | 15 | 13,15 | 7,8 | 20,23 | 10,11 | 15,16 | 11,17.3 | 18.3,21 | 21,23.2 | 10,12 | 11 | 8,12 | 11,12 | 8,14 |
| HOAZ_1005 | 13,15 | 16,19 | 9,12 | 17,18 | X,Y | 10,16 | 28,32.2 | 14,15 | 11,15 | 13,14 | 9,3 | 17,23 | 10,11 | 14,15 | 13,16 | 18,19 | 28.2,29.2 | 8,11 | 10,11 | 8 | 12,14 | 9,13 |
| HOAZ_1006 | 13,15 | 16,19 | 9,12 | 17,18 | X,Y | 10,16 | 28,32.2 | 14,15 | 11,15 | 13,14 | 9,3 | 17,23 | 10,11 | 14,15 | 13,16 | 18,19 | 28.2,29.2 | 8,11 | 10,11 | 8 | 12,14 | 9,13 |
| HOAZ_1010 | 14,16 | 14,18 | 9,11 | 16,17 | X | 12 | 28,29 | 14,18 | 16 | 15.2,16 | 6,9.3 | 21,25 | 11,12 | 16,17 | 11,14 | 20,21 | 17,2 | 10,12 | 10 | 8 | 11,12 | 11,12 |
| HOAZ_1011 | 14,16 | 14,18 | 9,11 | 16,17 | X | 12 | 28,29 | 14,18 | 16 | 15.2,16 | 6,9.3 | 21,25 | 11,12 | 16,17 | 11,14 | 20,21 | 17,2 | 10,12 | 10 | 8 | 11,12 | 11,12 |
| HOAZ_1012 | 15, 17 | 17, 18 | 9, 14 | 19, 24 | X, Y | 12, 16 | 27, 29 | 12, 14 | 15 | 13.2, 15.2 | 9 | 20, 24 | 11 | 15, 16 | 15, 17.3 | 17 | 28.2,29.2 | 8, 9 | 12, 13 | 8 | 10 | 8, 10 |
| HOAZ_1013 | 15, 17 | 17, 18 | 9, 14 | 19, 24 | X, Y | 12, 16 | 27, 29 | 12, 14 | 15 | 13.2, 15.2 | 9 | 20, 24 | 11 | 15, 16 | 15, 17.3 | 17 | 28.2,29.2 | 8, 9 | 12, 13 | 8 | 10 | 8, 10 |
| HOAZ_1015 | 15, 17 | 16, 18 | 11 | 16, 20 | X | 14, 15 | 29, 29.2 | 12 | 14, 16 | 13 | 6, 8 | 19, 20 | 11, 12 | 15, 17 | 16, 17.3 | 19 | 31.2, 33.2 | 10 | 11, 14 | 8, 11 | 12 | 11, 12 |
| HOAZ_1016 | 15, 17 | 16, 18 | 11 | 16, 20 | X | 14, 15 | 29, 29.2 | 12 | 14, 16 | 13 | 6, 8 | 19, 20 | 11, 12 | 15, 17 | 16, 17.3 | 19 | 31.2, 33.2 | 10 | 11, 14 | 8, 11 | 12 | 11, 12 |
| HOAZ_1019 | 12, 15 | 14, 16 | 11, 14 | 19, 22 | X | 12, 13 | 30, 31 | 11, 17 | 15, 16 | 15, 15.2 | 6, 7 | 19, 22 | 10, 15 | 15 | 11, 15 | 15, 22 | 15, 31.2 | 8, 12 | 10, 12 | 8, 11 | 11, 12 | 8, 13 |
| HOAZ_1020 | 12, 15 | 14, 16 | 11, 14 | 19, 22 | X | 12, 13 | 30, 31 | 11, 17 | 15, 16 | 15, 15.2 | 6, 7 | 19, 22 | 10, 15 | 15 | 11, 15 | 15, 22 | 15, 31.2 | 8, 12 | 10, 12 | 8, 11 | 11, 12 | 8, 13 |
| HOAZ_1023 | 14, 16 | 15, 17 | 11, 13 | 19, 21 | X, Y | 10,14 | 28, 30 | 13, 16 | 14, 16 | 14, 15.2 | 6 | 23.2, 24 | 11, 14 | 14, 15 | 15, 15.3 | 18.3, 23 | 18, 22.2 | 9, 10 | 11 | 8, 11 | 11, 13 | 10, 11 |
| HOAZ_1024 | 14, 16 | 15, 17 | 11, 13 | 19, 21 | X, Y | 10,14 | 28, 30 | 13, 16 | 14, 16 | 14, 15.2 | 6 | 23.2, 24 | 11, 14 | 14, 15 | 15, 15.3 | 18.3, 23 | 18, 22.2 | 9, 10 | 11 | 8, 11 | 11, 13 | 10, 11 |
| HOAZ_1027 | 14, 15 | 14, 16 | 11, 12 | 17, 19 | X | 12, 13 | 29, 30 | 13 | 11, 15 | 14, 16.2 | 6, 9.3 | 24, 25 | 10, 11 | 16 | 16, 16.3 | 18, 19 | 15, 17 | 9, 11 | 12, 13 | 9, 11 | 12, 13 | 11 |
| HOAZ_1028 | 14, 15 | 14, 16 | 11, 12 | 17, 19 | X | 12, 13 | 29, 30 | 13 | 11, 15 | 14, 16.2 | 6, 9.3 | 24, 25 | 10, 11 | 16 | 16, 16.3 | 18, 19 | 15, 17 | 9, 11 | 12, 13 | 9, 11 | 12, 13 | 11 |
| HOAZ_1033 | 13,16 | 18,19 | 9,11 | 16,23 | X | 12,13 | 27,32.2 | 15,19 | 11,15 | 14 | 6,7 | 22,24 | 11,12 | 17,18 | 14,16 | 24,25 | 24.2,26.2 | 9,12 | 11 | 8 | 12,13 | 9,12 |
| HOAZ_1034 | 13,16 | 18,19 | 9,11 | 16,23 | X | 12,13 | 27,32.2 | 15,19 | 11,15 | 14 | 6,7 | 22,24 | 11,12 | 17,18 | 14,16 | 24,25 | 24.2,26.2 | 9,12 | 11 | 8 | 12,13 | 9,12 |
| HOAZ_1035 | 13, 15 | 17 | 11, 13 | 17, 21 | X | 10, 14 | 29, 30 | 12, 16 | 15, 16 | 15, 16.2 | 9,3 | 22, 25 | 11 | 15, 17 | 13, 17.3 | 18, 22 | 16, 20 | 8, 10 | 11, 12 | 8, 9 | 11 | 11 |
| HOAZ_1036 | 13, 15 | 17 | 11, 13 | 17, 21 | X | 10, 14 | 29, 30 | 12, 16 | 15, 16 | 15, 16.2 | 9,3 | 22, 25 | 11 | 15, 17 | 13, 17.3 | 18, 22 | 16, 20 | 8, 10 | 11, 12 | 8, 9 | 11 | 11 |
| HOAZ_1039 | 14,15 | 14,18 | 10,12 | 20,23 | X | 10,12 | 30 | 12 | 11,16 | 13,14 | 7,9.3 | 25,27 | 11,14 | 17,18 | 14,17 | 22,23 | 16,23.2 | 11,12 | 11,12 | 8 | 12,13 | 11 |
| HOAZ_1040 | 14,15 | 14,18 | 10,12 | 20,23 | X | 10,12 | 30 | 12 | 11,16 | 13,14 | 7,9.3 | 25,27 | 11,14 | 17,18 | 14,17 | 22,23 | 16,23.2 | 11,12 | 11,12 | 8 | 12,13 | 11 |
| HOAZ_1041 | 13, 16 | 14, 16 | 11, 12 | 18 | X, Y | 13 | 30, 30.2 | 12, 16 | 15, 16 | 13, 14 | 9 | 23 | 11.3, 13.3 | 16 | 16, 17 | 18 | 18, 29.2 | 8, 10 | 10 | 9, 12 | 11, 12 | 12, 13 |
| HOAZ_1042 | 13, 16 | 14, 16 | 11, 12 | 18 | X, Y | 13 | 30, 30.2 | 12, 16 | 15, 16 | 13, 14 | 9 | 23 | 11.3, 13.3 | 16 | 16, 17 | 18 | 18, 29.2 | 8, 10 | 10 | 9, 12 | 11, 12 | 12, 13 |
