# Supplementary material for: Micro-RNA signatures in monozygotic twins discordant for congenital heart defects
Source: PLoS One. 2019 Dec 5;14(12):e0226164. doi: 10.1371/journal.pone.0226164 (PMC6894838; doi:10.1371/journal.pone.0226164)
Supplement: S2 Table — (DOCX) [file pone.0226164.s002.docx]

**S2 Table:** Differentially abundant miRNAs in the blood of monozygotic twin pairs with CHD compared to their healthy co-twins as determined by microarry analysis (p<0.05).

| **miRNAs** | **Median Healthy** | **Median CHDs** | **STDV Healthy** | **STDV CHDs** | **Fold Change** | **log(2) fold change** | **P-value** | **AUC** | **Coefficient of Variation Healthy** | **Coefficient of Variation CHDs** | **Difference of Coefficient of Variations** |
| --- | --- | --- | --- | --- | --- | --- | --- | --- | --- | --- | --- |
| hsa-miR-5189-3p | 4,33 | 10,39 | 3,48 | 4,01 | 0,42 | -1,26 | 0,0176 | 0,77 | 0,58 | 0,43 | 0,15 |
| hsa-miR-4707-3p | 2,64 | 4,42 | 1,37 | 1,81 | 0,60 | -0,74 | 0,0208 | 0,66 | 0,44 | 0,44 | 0,00 |
| hsa-miR-1306-5p | 4,73 | 7,63 | 2,30 | 2,81 | 0,62 | -0,69 | 0,0206 | 0,67 | 0,43 | 0,42 | 0,01 |
| hsa-miR-4732-3p | 34,81 | 53,13 | 25,02 | 36,38 | 0,66 | -0,61 | 0,0102 | 0,64 | 0,58 | 0,62 | -0,04 |
| hsa-miR-3605-3p | 3,62 | 5,22 | 1,19 | 1,42 | 0,69 | -0,53 | 0,0447 | 0,66 | 0,30 | 0,30 | 0,00 |
| hsa-miR-550b-2-5p | 5,62 | 7,69 | 2,22 | 2,15 | 0,73 | -0,45 | 0,0228 | 0,65 | 0,38 | 0,31 | 0,07 |
| hsa-miR-511-3p | 0,83 | 1,13 | 0,23 | 0,29 | 0,74 | -0,44 | 0,0329 | 0,73 | 0,26 | 0,26 | 0,00 |
| hsa-miR-890 | 0,87 | 1,17 | 0,28 | 0,18 | 0,74 | -0,43 | 0,0309 | 0,81 | 0,30 | 0,16 | 0,15 |
| hsa-miR-671-5p | 2,47 | 3,31 | 0,95 | 1,16 | 0,75 | -0,42 | 0,0385 | 0,69 | 0,32 | 0,33 | 0,00 |
| hsa-miR-128-1-5p | 2,10 | 2,82 | 0,81 | 0,73 | 0,74 | -0,42 | 0,0483 | 0,72 | 0,34 | 0,26 | 0,08 |
| hsa-miR-421 | 2,54 | 3,35 | 0,76 | 0,67 | 0,76 | -0,40 | 0,0193 | 0,72 | 0,28 | 0,21 | 0,07 |
| hsa-miR-129-2-3p | 4,04 | 5,27 | 1,10 | 1,00 | 0,77 | -0,39 | 0,0102 | 0,65 | 0,26 | 0,21 | 0,05 |
| hsa-miR-4420 | 2,56 | 3,34 | 0,72 | 0,71 | 0,77 | -0,38 | 0,0263 | 0,68 | 0,27 | 0,23 | 0,04 |
| hsa-miR-8088 | 0,87 | 1,12 | 0,20 | 0,22 | 0,78 | -0,36 | 0,0139 | 0,80 | 0,24 | 0,22 | 0,03 |
| hsa-miR-1270 | 2,17 | 2,78 | 1,32 | 1,58 | 0,78 | -0,36 | 0,0151 | 0,68 | 0,47 | 0,46 | 0,01 |
| hsa-miR-4499 | 3,92 | 5,02 | 0,92 | 1,04 | 0,78 | -0,36 | 0,0367 | 0,69 | 0,23 | 0,22 | 0,01 |
| hsa-miR-1178-5p | 0,78 | 0,99 | 0,20 | 0,27 | 0,79 | -0,34 | 0,0105 | 0,71 | 0,26 | 0,28 | -0,02 |
| hsa-miR-6716-5p | 0,74 | 0,94 | 0,19 | 0,19 | 0,79 | -0,34 | 0,0219 | 0,78 | 0,28 | 0,21 | 0,06 |
| hsa-miR-6888-5p | 1,00 | 1,27 | 0,17 | 0,19 | 0,79 | -0,33 | 0,0102 | 0,87 | 0,17 | 0,14 | 0,03 |
| hsa-miR-3163 | 2,49 | 3,12 | 0,71 | 0,72 | 0,80 | -0,33 | 0,0383 | 0,65 | 0,28 | 0,25 | 0,04 |
| hsa-miR-320d | 807,48 | 1013,03 | 677,18 | 938,12 | 0,80 | -0,33 | 0,0454 | 0,60 | 0,67 | 0,74 | -0,07 |
| hsa-miR-4451 | 1,22 | 1,52 | 0,22 | 0,27 | 0,80 | -0,32 | 0,0138 | 0,81 | 0,17 | 0,17 | 0,01 |
| hsa-miR-3923 | 2,10 | 2,60 | 0,36 | 0,36 | 0,81 | -0,31 | 0,0189 | 0,76 | 0,16 | 0,14 | 0,02 |
| hsa-miR-6882-5p | 0,74 | 0,92 | 0,13 | 0,20 | 0,81 | -0,31 | 0,0204 | 0,73 | 0,16 | 0,22 | -0,06 |
| hsa-miR-4676-5p | 2,07 | 2,55 | 0,68 | 0,62 | 0,81 | -0,30 | 0,0017 | 0,71 | 0,31 | 0,24 | 0,07 |
| hsa-miR-5009-3p | 1,59 | 1,95 | 0,26 | 0,35 | 0,81 | -0,30 | 0,0129 | 0,71 | 0,17 | 0,19 | -0,02 |
| hsa-miR-744-5p | 14,07 | 17,19 | 7,93 | 8,56 | 0,82 | -0,29 | 0,0171 | 0,62 | 0,57 | 0,49 | 0,08 |
| hsa-miR-3130-5p | 1,59 | 1,95 | 0,30 | 0,33 | 0,82 | -0,29 | 0,0207 | 0,68 | 0,18 | 0,18 | 0,01 |
| hsa-miR-1237-5p | 1,00 | 1,21 | 0,30 | 0,23 | 0,82 | -0,28 | 0,0397 | 0,68 | 0,27 | 0,19 | 0,09 |
| hsa-miR-4784 | 1,61 | 1,94 | 0,23 | 0,28 | 0,83 | -0,27 | 0,0083 | 0,81 | 0,14 | 0,14 | 0,00 |
| hsa-miR-5010-5p | 2,11 | 2,55 | 1,29 | 1,64 | 0,83 | -0,27 | 0,0125 | 0,67 | 0,51 | 0,52 | -0,01 |
| hsa-miR-7515 | 1,00 | 1,20 | 0,27 | 0,22 | 0,83 | -0,27 | 0,0265 | 0,72 | 0,25 | 0,17 | 0,07 |
| hsa-miR-5584-5p | 0,95 | 1,14 | 0,22 | 0,21 | 0,83 | -0,27 | 0,0328 | 0,72 | 0,24 | 0,19 | 0,05 |
| hsa-miR-3609 | 1,70 | 2,05 | 0,27 | 0,34 | 0,83 | -0,27 | 0,0442 | 0,72 | 0,15 | 0,17 | -0,02 |
| hsa-miR-139-3p | 4,08 | 4,86 | 1,02 | 1,14 | 0,84 | -0,25 | 0,0096 | 0,68 | 0,23 | 0,23 | 0,00 |
| hsa-miR-186-3p | 2,05 | 2,44 | 0,36 | 0,31 | 0,84 | -0,25 | 0,0264 | 0,74 | 0,18 | 0,13 | 0,05 |
| hsa-miR-3188 | 1,61 | 1,91 | 0,44 | 0,39 | 0,84 | -0,25 | 0,0294 | 0,68 | 0,27 | 0,21 | 0,06 |
| hsa-miR-6134 | 1,33 | 1,58 | 0,58 | 0,41 | 0,84 | -0,25 | 0,0457 | 0,65 | 0,39 | 0,24 | 0,15 |
| hsa-miR-3912-3p | 1,81 | 2,14 | 0,41 | 0,41 | 0,84 | -0,24 | 0,0301 | 0,67 | 0,23 | 0,20 | 0,03 |
| hsa-miR-548ba | 2,25 | 2,65 | 0,33 | 0,25 | 0,85 | -0,24 | 0,0482 | 0,69 | 0,14 | 0,10 | 0,05 |
| hsa-miR-4719 | 1,97 | 2,31 | 0,25 | 0,35 | 0,85 | -0,23 | 0,0004 | 0,83 | 0,13 | 0,15 | -0,02 |
| hsa-miR-3171 | 2,45 | 2,88 | 0,31 | 0,45 | 0,85 | -0,23 | 0,0024 | 0,73 | 0,13 | 0,17 | -0,04 |
| hsa-miR-448 | 1,96 | 2,30 | 0,24 | 0,33 | 0,85 | -0,23 | 0,0147 | 0,68 | 0,12 | 0,15 | -0,03 |
| hsa-miR-3919 | 1,04 | 1,22 | 0,27 | 0,24 | 0,85 | -0,23 | 0,0243 | 0,66 | 0,25 | 0,20 | 0,05 |
| hsa-miR-1827 | 1,14 | 1,34 | 0,21 | 0,17 | 0,85 | -0,23 | 0,0466 | 0,77 | 0,19 | 0,13 | 0,06 |
| hsa-miR-4253 | 1,59 | 1,85 | 0,22 | 0,22 | 0,86 | -0,22 | 0,0086 | 0,75 | 0,14 | 0,12 | 0,02 |
| hsa-miR-4753-5p | 1,50 | 1,75 | 0,38 | 0,42 | 0,86 | -0,22 | 0,0157 | 0,73 | 0,26 | 0,23 | 0,03 |
| hsa-miR-4760-5p | 1,23 | 1,44 | 0,26 | 0,28 | 0,86 | -0,22 | 0,0162 | 0,70 | 0,21 | 0,20 | 0,01 |
| hsa-miR-6761-5p | 1,11 | 1,30 | 0,15 | 0,24 | 0,86 | -0,22 | 0,0451 | 0,81 | 0,14 | 0,19 | -0,05 |
| hsa-miR-8067 | 1,65 | 1,91 | 0,28 | 0,27 | 0,87 | -0,21 | 0,0110 | 0,76 | 0,18 | 0,14 | 0,03 |
| hsa-miR-5697 | 1,49 | 1,72 | 0,16 | 0,25 | 0,86 | -0,21 | 0,0446 | 0,71 | 0,11 | 0,15 | -0,04 |
| hsa-miR-181b-3p | 1,32 | 1,52 | 0,19 | 0,13 | 0,87 | -0,20 | 0,0078 | 0,83 | 0,14 | 0,09 | 0,06 |
| hsa-miR-361-3p | 145,98 | 166,92 | 51,24 | 60,86 | 0,87 | -0,19 | 0,0151 | 0,60 | 0,39 | 0,40 | -0,01 |
| hsa-miR-6844 | 2,60 | 2,95 | 0,37 | 0,41 | 0,88 | -0,19 | 0,0199 | 0,70 | 0,14 | 0,14 | 0,00 |
| hsa-miR-184 | 1,54 | 1,76 | 0,16 | 0,30 | 0,88 | -0,19 | 0,0347 | 0,71 | 0,10 | 0,17 | -0,07 |
| hsa-miR-92a-1-5p | 1,40 | 1,59 | 0,14 | 0,17 | 0,88 | -0,19 | 0,0477 | 0,78 | 0,10 | 0,11 | -0,01 |
| hsa-miR-3610 | 1,34 | 1,52 | 0,27 | 0,32 | 0,88 | -0,19 | 0,0479 | 0,68 | 0,20 | 0,20 | 0,00 |
| hsa-miR-2053 | 2,90 | 3,30 | 0,38 | 0,41 | 0,88 | -0,18 | 0,0068 | 0,72 | 0,13 | 0,13 | 0,00 |
| hsa-miR-4480 | 1,83 | 2,08 | 0,23 | 0,33 | 0,88 | -0,18 | 0,0075 | 0,76 | 0,13 | 0,16 | -0,04 |
| hsa-miR-6880-5p | 1,70 | 1,93 | 0,25 | 0,25 | 0,88 | -0,18 | 0,0085 | 0,72 | 0,15 | 0,13 | 0,02 |
| hsa-miR-1252-3p | 2,80 | 3,16 | 0,34 | 0,42 | 0,88 | -0,18 | 0,0327 | 0,75 | 0,12 | 0,13 | -0,01 |
| hsa-miR-10b-3p | 1,79 | 2,03 | 0,32 | 0,29 | 0,89 | -0,18 | 0,0355 | 0,72 | 0,18 | 0,14 | 0,04 |
| hsa-miR-219a-5p | 2,27 | 2,56 | 0,29 | 0,29 | 0,89 | -0,17 | 0,0001 | 0,72 | 0,13 | 0,12 | 0,01 |
| hsa-miR-380-3p | 2,55 | 2,87 | 0,35 | 0,26 | 0,89 | -0,17 | 0,0082 | 0,72 | 0,14 | 0,10 | 0,04 |
| hsa-miR-125a-3p | 3,69 | 4,13 | 0,80 | 0,89 | 0,89 | -0,16 | 0,0020 | 0,67 | 0,22 | 0,21 | 0,01 |
| hsa-miR-1324 | 2,28 | 2,54 | 0,34 | 0,31 | 0,90 | -0,16 | 0,0136 | 0,78 | 0,15 | 0,12 | 0,03 |
| hsa-miR-3118 | 1,92 | 2,14 | 0,35 | 0,36 | 0,90 | -0,16 | 0,0160 | 0,65 | 0,19 | 0,17 | 0,02 |
| hsa-miR-539-3p | 2,24 | 2,49 | 0,49 | 0,33 | 0,90 | -0,15 | 0,0105 | 0,70 | 0,23 | 0,14 | 0,10 |
| hsa-miR-6876-5p | 2,54 | 2,83 | 0,39 | 0,35 | 0,90 | -0,15 | 0,0283 | 0,76 | 0,16 | 0,13 | 0,03 |
| hsa-miR-548f-3p | 2,30 | 2,56 | 0,39 | 0,39 | 0,90 | -0,15 | 0,0325 | 0,74 | 0,17 | 0,15 | 0,02 |
| hsa-miR-3679-3p | 3,48 | 3,84 | 0,40 | 0,50 | 0,91 | -0,14 | 0,0043 | 0,72 | 0,12 | 0,13 | -0,01 |
| hsa-miR-4696 | 1,76 | 1,95 | 0,20 | 0,30 | 0,91 | -0,14 | 0,0149 | 0,81 | 0,12 | 0,15 | -0,04 |
| hsa-miR-3202 | 1,73 | 1,92 | 0,31 | 0,32 | 0,90 | -0,14 | 0,0221 | 0,69 | 0,19 | 0,17 | 0,02 |
| hsa-miR-579-3p | 1,81 | 2,00 | 0,29 | 0,30 | 0,91 | -0,14 | 0,0242 | 0,73 | 0,17 | 0,15 | 0,02 |
| hsa-miR-4670-3p | 1,76 | 1,94 | 0,17 | 0,25 | 0,91 | -0,14 | 0,0245 | 0,66 | 0,10 | 0,13 | -0,03 |
| hsa-miR-5003-3p | 1,85 | 2,03 | 0,28 | 0,21 | 0,91 | -0,14 | 0,0316 | 0,76 | 0,15 | 0,10 | 0,05 |
| hsa-miR-496 | 1,86 | 2,04 | 0,23 | 0,29 | 0,91 | -0,14 | 0,0338 | 0,69 | 0,12 | 0,14 | -0,02 |
| hsa-miR-376a-2-5p | 2,43 | 2,66 | 0,27 | 0,33 | 0,91 | -0,14 | 0,0449 | 0,69 | 0,11 | 0,12 | -0,01 |
| hsa-miR-6507-5p | 1,58 | 1,72 | 0,32 | 0,20 | 0,91 | -0,13 | 0,0433 | 0,63 | 0,20 | 0,12 | 0,09 |
| hsa-miR-501-3p | 15,35 | 16,71 | 6,00 | 7,60 | 0,92 | -0,12 | 0,0309 | 0,62 | 0,43 | 0,45 | -0,01 |
| hsa-miR-4488 | 1,62 | 1,76 | 0,31 | 0,40 | 0,92 | -0,12 | 0,0377 | 0,67 | 0,18 | 0,22 | -0,03 |
| hsa-miR-548f-5p | 1,86 | 2,01 | 0,42 | 0,41 | 0,92 | -0,11 | 0,0029 | 0,64 | 0,23 | 0,20 | 0,03 |
| hsa-miR-676-3p | 1,44 | 1,54 | 0,19 | 0,24 | 0,93 | -0,11 | 0,0056 | 0,72 | 0,14 | 0,15 | -0,01 |
| hsa-miR-95-3p | 1,85 | 1,99 | 0,25 | 0,16 | 0,93 | -0,11 | 0,0103 | 0,72 | 0,14 | 0,08 | 0,06 |
| hsa-miR-7106-5p | 2,43 | 2,61 | 0,40 | 0,44 | 0,93 | -0,11 | 0,0129 | 0,72 | 0,17 | 0,16 | 0,01 |
| hsa-miR-497-5p | 1,86 | 2,00 | 0,22 | 0,23 | 0,93 | -0,11 | 0,0326 | 0,78 | 0,12 | 0,11 | 0,00 |
| hsa-miR-6506-5p | 1,56 | 1,67 | 0,17 | 0,19 | 0,93 | -0,10 | 0,0028 | 0,76 | 0,11 | 0,11 | 0,00 |
| hsa-miR-1256 | 2,27 | 2,43 | 0,29 | 0,14 | 0,93 | -0,10 | 0,0057 | 0,79 | 0,13 | 0,06 | 0,07 |
| hsa-miR-1323 | 2,47 | 2,64 | 0,27 | 0,39 | 0,93 | -0,10 | 0,0131 | 0,73 | 0,11 | 0,14 | -0,03 |
| hsa-miR-4638-5p | 1,10 | 1,18 | 0,18 | 0,21 | 0,93 | -0,10 | 0,0385 | 0,66 | 0,17 | 0,17 | -0,01 |
| hsa-miR-155-3p | 2,17 | 2,33 | 0,30 | 0,32 | 0,93 | -0,10 | 0,0425 | 0,68 | 0,14 | 0,14 | 0,00 |
| hsa-miR-3616-3p | 3,49 | 3,75 | 0,73 | 0,77 | 0,93 | -0,10 | 0,0442 | 0,66 | 0,21 | 0,20 | 0,01 |
| hsa-miR-3606-5p | 2,13 | 2,28 | 0,23 | 0,24 | 0,93 | -0,10 | 0,0499 | 0,69 | 0,11 | 0,11 | 0,00 |
| hsa-miR-19b-2-5p | 2,37 | 2,52 | 0,25 | 0,30 | 0,94 | -0,09 | 0,0095 | 0,76 | 0,11 | 0,12 | -0,01 |
| hsa-miR-548at-5p | 1,86 | 1,98 | 0,21 | 0,27 | 0,94 | -0,09 | 0,0499 | 0,71 | 0,11 | 0,13 | -0,02 |
| hsa-miR-3134 | 1,86 | 1,96 | 0,34 | 0,29 | 0,95 | -0,08 | 0,0048 | 0,68 | 0,19 | 0,14 | 0,05 |
| hsa-miR-4532 | 3,65 | 3,86 | 0,58 | 0,59 | 0,95 | -0,08 | 0,0149 | 0,70 | 0,17 | 0,15 | 0,02 |
| hsa-miR-1295a | 1,22 | 1,27 | 0,37 | 0,46 | 0,96 | -0,06 | 0,0237 | 0,62 | 0,29 | 0,32 | -0,03 |
| hsa-miR-95-5p | 2,01 | 2,08 | 0,31 | 0,25 | 0,97 | -0,05 | 0,0215 | 0,67 | 0,16 | 0,12 | 0,04 |
| hsa-miR-545-3p | 2,49 | 2,57 | 0,30 | 0,22 | 0,97 | -0,04 | 0,0282 | 0,63 | 0,12 | 0,09 | 0,04 |
| hsa-miR-153-3p | 1,23 | 1,27 | 0,21 | 0,29 | 0,98 | -0,04 | 0,0284 | 0,62 | 0,18 | 0,22 | -0,04 |
| hsa-miR-555 | 1,60 | 1,51 | 0,10 | 0,12 | 1,06 | 0,08 | 0,0107 | 0,27 | 0,06 | 0,08 | -0,01 |
| hsa-miR-521 | 2,58 | 2,44 | 0,58 | 0,29 | 1,06 | 0,08 | 0,0309 | 0,32 | 0,20 | 0,12 | 0,09 |
| hsa-miR-891a-5p | 1,63 | 1,54 | 0,30 | 0,25 | 1,06 | 0,08 | 0,0433 | 0,34 | 0,18 | 0,17 | 0,02 |
| hsa-miR-130a-5p | 2,23 | 2,10 | 0,32 | 0,24 | 1,06 | 0,09 | 0,0138 | 0,33 | 0,14 | 0,11 | 0,02 |
| hsa-miR-6849-3p | 2,13 | 2,00 | 1,25 | 0,96 | 1,06 | 0,09 | 0,0455 | 0,39 | 0,46 | 0,42 | 0,04 |
| hsa-miR-6890-3p | 3,39 | 3,18 | 1,81 | 1,22 | 1,06 | 0,09 | 0,0456 | 0,38 | 0,44 | 0,34 | 0,10 |
| hsa-miR-6854-3p | 1,90 | 1,78 | 0,15 | 0,22 | 1,07 | 0,10 | 0,0126 | 0,26 | 0,08 | 0,12 | -0,05 |
| hsa-miR-770-5p | 1,80 | 1,68 | 0,28 | 0,21 | 1,07 | 0,10 | 0,0422 | 0,33 | 0,15 | 0,12 | 0,03 |
| hsa-miR-429 | 1,70 | 1,58 | 0,16 | 0,22 | 1,07 | 0,10 | 0,0444 | 0,22 | 0,09 | 0,14 | -0,05 |
| hsa-miR-548v | 2,55 | 2,36 | 0,51 | 0,29 | 1,08 | 0,11 | 0,0067 | 0,27 | 0,19 | 0,12 | 0,06 |
| hsa-miR-605-5p | 2,74 | 2,55 | 0,79 | 0,25 | 1,08 | 0,11 | 0,0187 | 0,19 | 0,26 | 0,10 | 0,15 |
| hsa-miR-4750-3p | 3,18 | 2,93 | 0,41 | 0,34 | 1,09 | 0,12 | 0,0167 | 0,29 | 0,13 | 0,12 | 0,01 |
| hsa-miR-7114-3p | 3,78 | 3,48 | 4,18 | 2,06 | 1,09 | 0,12 | 0,0187 | 0,31 | 0,69 | 0,52 | 0,17 |
| hsa-miR-143-5p | 2,00 | 1,84 | 0,19 | 0,19 | 1,08 | 0,12 | 0,0363 | 0,31 | 0,10 | 0,10 | 0,00 |
| hsa-miR-4697-3p | 2,80 | 2,59 | 0,73 | 0,26 | 1,08 | 0,12 | 0,0417 | 0,30 | 0,24 | 0,10 | 0,14 |
| hsa-miR-34b-3p | 3,04 | 2,79 | 1,21 | 0,69 | 1,09 | 0,12 | 0,0446 | 0,35 | 0,35 | 0,24 | 0,12 |
| hsa-miR-6843-3p | 2,02 | 1,86 | 0,23 | 0,20 | 1,08 | 0,12 | 0,0464 | 0,26 | 0,11 | 0,11 | 0,00 |
| hsa-miR-3689b-3p | 1,51 | 1,37 | 0,28 | 0,25 | 1,10 | 0,14 | 0,0192 | 0,29 | 0,18 | 0,18 | 0,00 |
| hsa-miR-6785-3p | 4,35 | 3,95 | 5,18 | 2,97 | 1,10 | 0,14 | 0,0357 | 0,42 | 0,76 | 0,61 | 0,14 |
| hsa-miR-6868-5p | 1,19 | 1,07 | 0,30 | 0,30 | 1,10 | 0,14 | 0,0492 | 0,31 | 0,23 | 0,27 | -0,04 |
| hsa-miR-432-3p | 2,44 | 2,19 | 0,46 | 0,19 | 1,11 | 0,15 | 0,0102 | 0,18 | 0,18 | 0,09 | 0,09 |
| hsa-miR-99b-3p | 1,50 | 1,35 | 0,44 | 0,40 | 1,11 | 0,15 | 0,0351 | 0,37 | 0,27 | 0,28 | -0,01 |
| hsa-miR-3972 | 1,51 | 1,36 | 0,29 | 0,24 | 1,11 | 0,16 | 0,0249 | 0,29 | 0,19 | 0,18 | 0,01 |
| hsa-miR-6775-3p | 4,08 | 3,65 | 9,55 | 7,70 | 1,12 | 0,16 | 0,0443 | 0,38 | 1,02 | 1,17 | -0,16 |
| hsa-miR-6818-3p | 2,19 | 1,94 | 0,45 | 0,32 | 1,13 | 0,17 | 0,0227 | 0,31 | 0,20 | 0,16 | 0,04 |
| hsa-miR-5591-3p | 2,42 | 2,15 | 0,59 | 0,29 | 1,12 | 0,17 | 0,0241 | 0,28 | 0,23 | 0,13 | 0,09 |
| hsa-miR-4523 | 1,08 | 0,96 | 0,29 | 0,19 | 1,13 | 0,17 | 0,0295 | 0,24 | 0,25 | 0,21 | 0,05 |
| hsa-miR-3650 | 1,72 | 1,53 | 0,35 | 0,24 | 1,12 | 0,17 | 0,0318 | 0,30 | 0,20 | 0,16 | 0,04 |
| hsa-miR-6808-3p | 1,51 | 1,35 | 0,47 | 0,26 | 1,12 | 0,17 | 0,0366 | 0,36 | 0,30 | 0,19 | 0,11 |
| hsa-miR-6778-3p | 2,04 | 1,82 | 0,31 | 0,25 | 1,12 | 0,17 | 0,0410 | 0,28 | 0,15 | 0,14 | 0,01 |
| hsa-miR-764 | 2,95 | 2,60 | 0,76 | 0,48 | 1,13 | 0,18 | 0,0164 | 0,26 | 0,24 | 0,18 | 0,06 |
| hsa-miR-1226-3p | 1,68 | 1,48 | 0,54 | 0,18 | 1,14 | 0,18 | 0,0216 | 0,15 | 0,28 | 0,12 | 0,16 |
| hsa-miR-92b-3p | 4,14 | 3,65 | 0,86 | 0,84 | 1,14 | 0,18 | 0,0226 | 0,33 | 0,20 | 0,22 | -0,02 |
| hsa-miR-4708-5p | 1,88 | 1,66 | 0,81 | 0,27 | 1,13 | 0,18 | 0,0453 | 0,22 | 0,37 | 0,16 | 0,21 |
| hsa-miR-1914-5p | 2,03 | 1,78 | 0,46 | 0,20 | 1,14 | 0,18 | 0,0477 | 0,24 | 0,22 | 0,11 | 0,10 |
| hsa-miR-6500-5p | 2,13 | 1,87 | 0,32 | 0,28 | 1,14 | 0,19 | 0,0293 | 0,29 | 0,15 | 0,14 | 0,00 |
| hsa-miR-1538 | 2,38 | 2,09 | 0,48 | 0,40 | 1,14 | 0,19 | 0,0317 | 0,30 | 0,19 | 0,18 | 0,01 |
| hsa-miR-4649-3p | 8,18 | 7,19 | 13,53 | 9,04 | 1,14 | 0,19 | 0,0342 | 0,36 | 0,98 | 0,96 | 0,01 |
| hsa-miR-487b-5p | 2,13 | 1,87 | 1,01 | 0,29 | 1,14 | 0,19 | 0,0354 | 0,31 | 0,39 | 0,15 | 0,25 |
| hsa-miR-5001-3p | 2,01 | 1,76 | 0,28 | 0,26 | 1,14 | 0,19 | 0,0479 | 0,27 | 0,14 | 0,14 | 0,00 |
| hsa-miR-300 | 1,87 | 1,63 | 0,21 | 0,21 | 1,15 | 0,20 | 0,0081 | 0,16 | 0,11 | 0,13 | -0,01 |
| hsa-miR-6852-3p | 2,31 | 2,01 | 0,73 | 0,20 | 1,15 | 0,20 | 0,0208 | 0,26 | 0,29 | 0,10 | 0,19 |
| hsa-miR-517c-3p | 2,22 | 1,93 | 0,28 | 0,31 | 1,15 | 0,20 | 0,0308 | 0,32 | 0,13 | 0,16 | -0,03 |
| hsa-miR-6839-5p | 1,56 | 1,35 | 0,34 | 0,31 | 1,16 | 0,21 | 0,0049 | 0,28 | 0,21 | 0,22 | -0,01 |
| hsa-miR-6822-3p | 2,25 | 1,94 | 0,46 | 0,24 | 1,16 | 0,21 | 0,0106 | 0,26 | 0,20 | 0,13 | 0,08 |
| hsa-miR-4745-3p | 1,06 | 0,91 | 0,13 | 0,12 | 1,16 | 0,21 | 0,0107 | 0,19 | 0,12 | 0,13 | -0,01 |
| hsa-miR-3160-5p | 2,93 | 2,52 | 0,60 | 0,45 | 1,16 | 0,21 | 0,0119 | 0,29 | 0,20 | 0,17 | 0,03 |
| hsa-miR-6827-3p | 2,49 | 2,15 | 0,44 | 0,28 | 1,16 | 0,21 | 0,0213 | 0,22 | 0,17 | 0,13 | 0,04 |
| hsa-miR-489-5p | 1,80 | 1,55 | 0,23 | 0,24 | 1,16 | 0,21 | 0,0432 | 0,36 | 0,13 | 0,15 | -0,01 |
| hsa-miR-2113 | 2,01 | 1,72 | 0,41 | 0,31 | 1,17 | 0,22 | 0,0262 | 0,33 | 0,20 | 0,18 | 0,02 |
| hsa-miR-3691-3p | 2,18 | 1,87 | 0,40 | 0,25 | 1,17 | 0,22 | 0,0280 | 0,24 | 0,18 | 0,13 | 0,05 |
| hsa-miR-6810-3p | 2,74 | 2,35 | 0,42 | 0,24 | 1,17 | 0,22 | 0,0351 | 0,22 | 0,15 | 0,10 | 0,05 |
| hsa-miR-6793-3p | 2,25 | 1,93 | 0,64 | 0,53 | 1,17 | 0,22 | 0,0388 | 0,35 | 0,27 | 0,26 | 0,01 |
| hsa-miR-1250-5p | 1,82 | 1,56 | 0,21 | 0,23 | 1,17 | 0,22 | 0,0400 | 0,26 | 0,12 | 0,15 | -0,03 |
| hsa-miR-6809-3p | 2,84 | 2,42 | 0,43 | 0,23 | 1,17 | 0,23 | 0,0062 | 0,19 | 0,15 | 0,10 | 0,05 |
| hsa-miR-6762-3p | 2,16 | 1,84 | 0,49 | 0,29 | 1,18 | 0,23 | 0,0120 | 0,33 | 0,22 | 0,16 | 0,07 |
| hsa-miR-585-5p | 1,53 | 1,30 | 0,26 | 0,37 | 1,18 | 0,23 | 0,0383 | 0,27 | 0,16 | 0,27 | -0,11 |
| hsa-miR-4747-3p | 2,00 | 1,71 | 0,40 | 0,32 | 1,17 | 0,23 | 0,0427 | 0,22 | 0,20 | 0,18 | 0,02 |
| hsa-miR-3191-5p | 2,23 | 1,89 | 0,48 | 0,21 | 1,18 | 0,24 | 0,0177 | 0,19 | 0,20 | 0,11 | 0,09 |
| hsa-miR-676-5p | 1,62 | 1,37 | 0,21 | 0,26 | 1,18 | 0,24 | 0,0205 | 0,26 | 0,13 | 0,18 | -0,05 |
| hsa-miR-4751 | 0,90 | 0,76 | 0,14 | 0,11 | 1,19 | 0,25 | 0,0027 | 0,16 | 0,15 | 0,15 | 0,00 |
| hsa-miR-4667-3p | 3,00 | 2,53 | 1,16 | 0,91 | 1,19 | 0,25 | 0,0117 | 0,32 | 0,36 | 0,33 | 0,02 |
| hsa-miR-4447 | 1,86 | 1,56 | 0,37 | 0,39 | 1,19 | 0,25 | 0,0489 | 0,36 | 0,21 | 0,26 | -0,05 |
| hsa-miR-597-5p | 1,65 | 1,37 | 0,24 | 0,24 | 1,20 | 0,26 | 0,0238 | 0,23 | 0,15 | 0,16 | -0,02 |
| hsa-miR-1199-3p | 1,36 | 1,13 | 0,47 | 0,27 | 1,21 | 0,27 | 0,0106 | 0,27 | 0,31 | 0,22 | 0,08 |
| hsa-miR-619-3p | 1,78 | 1,47 | 0,50 | 0,27 | 1,21 | 0,27 | 0,0143 | 0,23 | 0,26 | 0,18 | 0,08 |
| hsa-miR-6868-3p | 1,60 | 1,33 | 0,16 | 0,20 | 1,21 | 0,27 | 0,0170 | 0,17 | 0,10 | 0,15 | -0,05 |
| hsa-miR-3158-3p | 1,91 | 1,57 | 0,31 | 0,23 | 1,22 | 0,28 | 0,0272 | 0,19 | 0,16 | 0,14 | 0,02 |
| hsa-miR-4297 | 2,37 | 1,95 | 0,76 | 0,23 | 1,21 | 0,28 | 0,0383 | 0,28 | 0,31 | 0,12 | 0,19 |
| hsa-miR-4485-5p | 32,31 | 26,49 | 10,71 | 12,48 | 1,22 | 0,29 | 0,0292 | 0,28 | 0,30 | 0,42 | -0,11 |
| hsa-miR-889-5p | 1,72 | 1,40 | 0,23 | 0,15 | 1,23 | 0,30 | 0,0017 | 0,12 | 0,13 | 0,11 | 0,02 |
| hsa-miR-6729-5p | 0,87 | 0,70 | 0,14 | 0,25 | 1,23 | 0,30 | 0,0170 | 0,26 | 0,16 | 0,33 | -0,17 |
| hsa-miR-3619-3p | 2,06 | 1,67 | 0,55 | 0,46 | 1,23 | 0,30 | 0,0294 | 0,29 | 0,26 | 0,27 | -0,01 |
| hsa-miR-3944-3p | 1,39 | 1,13 | 0,39 | 0,37 | 1,23 | 0,30 | 0,0314 | 0,31 | 0,27 | 0,30 | -0,03 |
| hsa-miR-3192-3p | 2,73 | 2,21 | 0,71 | 0,49 | 1,24 | 0,31 | 0,0368 | 0,31 | 0,26 | 0,21 | 0,05 |
| hsa-miR-6730-3p | 4,34 | 3,47 | 3,21 | 1,66 | 1,25 | 0,32 | 0,0306 | 0,41 | 0,60 | 0,42 | 0,18 |
| hsa-miR-4701-5p | 6,02 | 4,84 | 6,93 | 3,31 | 1,24 | 0,32 | 0,0470 | 0,34 | 0,82 | 0,57 | 0,25 |
| hsa-miR-6085 | 29,49 | 23,47 | 22,96 | 14,27 | 1,26 | 0,33 | 0,0253 | 0,40 | 0,64 | 0,54 | 0,11 |
| hsa-miR-8070 | 1,55 | 1,23 | 0,37 | 0,23 | 1,26 | 0,33 | 0,0295 | 0,24 | 0,23 | 0,18 | 0,05 |
| hsa-miR-6804-5p | 1,08 | 0,85 | 0,27 | 0,21 | 1,27 | 0,34 | 0,0041 | 0,25 | 0,23 | 0,23 | 0,00 |
| hsa-miR-939-3p | 3,25 | 2,57 | 1,26 | 0,79 | 1,26 | 0,34 | 0,0203 | 0,35 | 0,36 | 0,28 | 0,08 |
| hsa-miR-4279 | 2,24 | 1,77 | 0,52 | 0,43 | 1,27 | 0,34 | 0,0449 | 0,33 | 0,25 | 0,24 | 0,01 |
| hsa-miR-6886-3p | 3,97 | 3,14 | 7,52 | 3,01 | 1,26 | 0,34 | 0,0467 | 0,35 | 1,02 | 0,75 | 0,27 |
| hsa-miR-6814-3p | 1,68 | 1,32 | 0,46 | 0,18 | 1,27 | 0,35 | 0,0222 | 0,19 | 0,26 | 0,13 | 0,13 |
| hsa-miR-4800-3p | 2,24 | 1,72 | 0,60 | 0,43 | 1,31 | 0,39 | 0,0158 | 0,32 | 0,27 | 0,23 | 0,04 |
| hsa-miR-3714 | 2,66 | 2,02 | 1,02 | 0,63 | 1,32 | 0,40 | 0,0043 | 0,21 | 0,35 | 0,28 | 0,07 |
| hsa-miR-6879-3p | 2,66 | 2,02 | 1,04 | 0,79 | 1,32 | 0,40 | 0,0250 | 0,38 | 0,37 | 0,34 | 0,03 |
| hsa-miR-675-3p | 2,42 | 1,84 | 0,67 | 0,69 | 1,32 | 0,40 | 0,0292 | 0,27 | 0,27 | 0,34 | -0,06 |
| hsa-miR-6743-3p | 5,02 | 3,81 | 13,28 | 8,08 | 1,32 | 0,40 | 0,0312 | 0,37 | 1,12 | 1,15 | -0,04 |
| hsa-miR-8075 | 1,59 | 1,20 | 0,57 | 0,29 | 1,33 | 0,41 | 0,0241 | 0,28 | 0,35 | 0,23 | 0,12 |
| hsa-miR-6727-3p | 2,08 | 1,55 | 0,57 | 0,66 | 1,34 | 0,42 | 0,0033 | 0,29 | 0,26 | 0,37 | -0,10 |
| hsa-miR-6736-3p | 3,14 | 2,34 | 0,84 | 0,56 | 1,34 | 0,42 | 0,0083 | 0,30 | 0,28 | 0,23 | 0,05 |
| hsa-miR-1343-3p | 2,42 | 1,80 | 0,89 | 0,71 | 1,35 | 0,43 | 0,0307 | 0,33 | 0,34 | 0,34 | 0,00 |
| hsa-miR-134-3p | 2,43 | 1,78 | 0,56 | 0,36 | 1,36 | 0,45 | 0,0064 | 0,26 | 0,24 | 0,19 | 0,05 |
| hsa-miR-7106-3p | 2,40 | 1,76 | 0,96 | 0,50 | 1,37 | 0,45 | 0,0086 | 0,23 | 0,36 | 0,25 | 0,11 |
| hsa-miR-6789-3p | 2,54 | 1,85 | 0,91 | 0,63 | 1,37 | 0,45 | 0,0366 | 0,35 | 0,35 | 0,30 | 0,05 |
| hsa-miR-1825 | 7,09 | 5,19 | 13,02 | 6,84 | 1,37 | 0,45 | 0,0481 | 0,35 | 1,07 | 0,90 | 0,18 |
| hsa-miR-4763-5p | 3,68 | 2,66 | 1,71 | 1,05 | 1,38 | 0,47 | 0,0360 | 0,36 | 0,45 | 0,36 | 0,09 |
| hsa-miR-3677-3p | 1,18 | 0,84 | 0,40 | 0,19 | 1,41 | 0,50 | 0,0083 | 0,24 | 0,34 | 0,21 | 0,13 |
| hsa-miR-4296 | 1,50 | 1,04 | 0,44 | 0,51 | 1,45 | 0,53 | 0,0459 | 0,36 | 0,30 | 0,41 | -0,11 |
| hsa-miR-1281 | 7,78 | 5,35 | 16,10 | 9,02 | 1,45 | 0,54 | 0,0299 | 0,33 | 1,10 | 1,04 | 0,06 |
| hsa-miR-6877-3p | 4,13 | 2,82 | 1,30 | 0,51 | 1,46 | 0,55 | 0,0090 | 0,23 | 0,33 | 0,17 | 0,16 |
| hsa-miR-6787-3p | 3,15 | 2,12 | 2,37 | 1,41 | 1,49 | 0,57 | 0,0133 | 0,35 | 0,63 | 0,52 | 0,10 |
| hsa-miR-6735-3p | 2,73 | 1,81 | 1,66 | 0,72 | 1,50 | 0,59 | 0,0329 | 0,24 | 0,53 | 0,34 | 0,19 |
| hsa-miR-6511a-3p | 4,38 | 2,76 | 4,38 | 1,52 | 1,59 | 0,66 | 0,0323 | 0,30 | 0,77 | 0,48 | 0,29 |
| hsa-miR-943 | 2,07 | 1,28 | 0,64 | 0,42 | 1,61 | 0,69 | 0,0038 | 0,24 | 0,32 | 0,29 | 0,03 |
| hsa-miR-7109-3p | 4,28 | 2,37 | 2,27 | 1,63 | 1,80 | 0,85 | 0,0278 | 0,38 | 0,56 | 0,56 | 0,00 |

CHDs, Congenital heart defects; AUC, Area Under the Curve
